# Medical and Philosophical Intelligence

**Published:** 1815-10

**Authors:** 


### MEDICAL AND PHILOSOPHICAL INTELLIGENCE.

### APOTHECARIES' ACT.

THE verbosity of Acts of Parliament is so irksome to medical readers, who look for nothing but matter of fact, that we have been at some pains to make a faithful abstract, well aware that the very fatigue of a minute perusal is enough to prevent the recollection of the most important clauses.

The preamble recites the patent of King James I. by which the apothecaries were separated from the grocers, and adds, that, whereas some of the clauses of the above charter have been found inadequate, his Majesty is, therefore, advised to enact, by Parliament,

1st, The confirmation of said charter, excepting as altered by this Act. By this they are empowered to examine all persons within seven miles of London who practise as apothecaries, and also their shops.

2d, That persons appointed by the Master, Wardens, and Society, or Court of Assistants, shall, at any hour of the day, have power to enter the shops of persons exercising the art and mystery of apothecaries, examine their contents, and when they find any thing improper, burn, or otherwise destroy, them, report them to the Master and Wardens, who, for the first offence, may fine them *5l.*; for the second *10l.*; for the third, and every future offence, *20l.*

The qualification of the persons to be appointed by the Master, Wardens, &c. are next specified. For examining shops in and within thirty miles of London, the persons appointed must have been ten years members of the Apothecaries' Company. For examining beyond those limits, the persons appointed by the said Master, &c. must have been a practising apothecary five years.

For refusing to compound medicines according to the directions of a licensed physician, the penalty, first *5l.*, second *10l.*, and third forfeiture of certificate.

Master and Wardens may appoint deputies.

The present Company, as chartered by James I. are appointed to carry the Act into execution.

The Master and Wardens can only act at meetings regularly called.

The Court of Examiners are to appoint a chairman, and to be sworn. At the end of the oath a clause is added, that the said

Master, Wardens, or Court of Assistants, or the major part of them, are to administer the oath.

The examiners retain their office for a year, unless removed by the Master and Wardens. They may be rechosen.

Persons already in practice are not required to be examined, but all others commencing from the 1st of August next, after passing the Act.

All persons examined must have attained their 21st year, have served five years' apprenticeship, and produce testimonials of having had the means of a medical education, and of having a good moral conduct.

Proper notice to be given by such as wish to be examined.

A court of five examiners is appointed by the same authority, for the examination of assistant-apothecaries not more than thirty miles from London.

The sums paid for a certificate to practise as an apothecary within ten miles of London, ten guineas; beyond that, six guineas. Penalty for acting as an apothecary without certificate, 20*l.* for each offence; as an assistant, 5*l.*

No one commencing practice after August, 1815, without a certificate, can recover charges for medicines or attendance.

Persons refused their certificate on account of incapacity under examination, may apply again.

An annual list is to be published of all who are licensed in that year, mentioning their residence.

All the money received for certificates to be paid into the hands of the Master and Wardens, to be disposed of as they shall direct and deem expedient.

Money from penalties goes half to the informer, and half to the Master and Wardens as above.

Two clauses follow, showing the manner in which the penalties are to be recovered.

The two following clauses seem to us likely to introduce some obscurity into the whole.

"Provided always, and be it further enacted, That nothing in this Act contained shall extend, or be construed to extend, to prejudice, or in any way to affect the trade or business of a chemist and druggist, in the buying, preparing, compounding, dispensing, and vending drugs, medicines, and medicinable compounds, wholesale and retail; but all persons using or exercising the said trade or business, or who shall or may hereafter use or exercise the same, shall and may use, exercise, and carry on the same trade or business, in such manner, and as fully and amply to all intents and purposes, as the same trade or business was used, exercised, or carried on by chemists and druggists before the passing of this Act.

"Provided always, and be it further enacted, That nothing in this Act contained shall extend, or be construed to extend, to lessen, prejudice, or defeat, or in any wise to interfere with any of the rights, authorities, privileges, and immunities heretofore vested in and exercised and enjoyed by either of the two Universities of Oxford or Cambridge, the Royal College of Physicians, the Royal College of Surgeons, or the said Society of Apothecaries respectively, other than and except such as shall or may have been altered, varied, or amended in and by this Act, or of any person or persons practising as an apothecary previously to the first day of August 1815; but the said Universities, Royal Colleges, and the said Society, and all such persons or person, shall have, use, exercise, and enjoy all such rights, authorities, privileges, and immunities, save and except as aforesaid, in as full, ample, and beneficial a manner, to all intents and purposes, as they might have done before the passing of this Act, and in case the same had never been passed."

The two concluding clauses limit the period of suits or actions, and make the bill a public act.

Such is the nature of an Act perhaps the most important that has ever been introduced into the practice of medicine. It must tend to render the education much more expensive, and, we trust, much more respectable. The present character of the Society of Apothecaries stands too high to need any comment, and the Examiners appointed by the Court are too well known, and too much respected, to admit of the slightest objection against them. The bill must be expected, like all other new laws, to require some modification. Among the rest, we would propose a few to the opinion of better judges.

First, we conceive that no person's indentures as an apprentice shall be hereafter valid, unless he produces with them a certificate from some authorised provincial schoolmaster or clergyman, given before he was apprenticed, that his knowledge of Latin is sufficient to construe what may be likely to be presented to him, at least with the assistance of a dictionary.

Next, that such persons as before the present Act were articled for less than five years, shall, after making up the remainder of the time as assistants, be admitted to an examination.

We conceive the clause relative to chemists and druggists too little defined, until the art and mystery of an apothecary is defined also.

As none of the clauses are to affect the rights of other chartered bodies, the present act cannot be perfectly comprehended, till the charters and rights of the two learned Colleges and Universities are defined.

Among the communications we have received on the subject, the two following are all we have room for at present.

*To the Editor of the London Medical and Physical Journal.*

Sir,—On perusing an Abstract of the Bill for the better regulating the education, &c. of apothecaries, I was somewhat surprised to find that it is intended to lengthen the apprenticeship of those whose indentures were executed previous to the present time. I cannot say, sir, that I am much pleased with this: I am an apprentice myself (to a surgeon-apothecary), and in the last year of my apprenticeship; now, my indentures bind me only for three years and and a half, but at the expiration of that time I shall have served upwards of four years, having been in my present situation nine months before these (the indentures) were executed, yet, by the new Act, I am to continue here a year and a half longer, to make up the specified time; on the whole, therefore, I must be confined six years.

This must necessarily shorten the time I had intended to devote to the attendance of the public hospitals and lectures, from which I hoped to derive more practical instruction than what I can receive here, and which I was the more earnest in my endeavours to procure, as it is my intention to combine the practice of the two professions, pharmacy and surgery.

As there must be many others in my situation, I think the best plan we can adopt will be to draw up a petition for the revision and amendment of certain sections in the new Act, whereby all those whose apprenticeship had commenced before the existence of the said Act, may be deemed sufficiently qualified to act as apothecaries or assistants after having passed a proper examination as now intended.

Perhaps this may meet the eye of some, either masters or apprentices, who may be enabled to propose some better plan, in hopes of which, I remain, sir, your's very respectfully,

*August* 15, 1815. A Pupil.

*To the Editor of the London Medical and Physical Journal.*

Sir,—The following observations relating to the Apothecaries' Bill are submitted to your consideration by *A Constant Reader.*

*August* 12, 1815.

As this Act professes to be prospective, I think it very severe, as well as unjust, that young men who have served an apprenticeship of three or four years, which may have expired before this Act passed, are not allowed to pass an examination at Apothecaries' Hall without being bound again, which might prove extremely inconvenient, as it may interfere with their studies in London. Neither ought this Act to interfere with people now under Indentures, as unprincipled men might take advantage of their situation, and demand an exorbitant premium for the prolongation of the term.

Surely it is not right that those who have passed, previous to this Act, the Royal College of Surgeons, and have been distinguished both by abilities and industry, are not allowed to pass an examination in order to practise as an apothecary, because they have not completed an apprenticeship of five years.

These grievances, Mr. Editor, are not imaginary, for, even in my small number of friends, I am acquainted with individuals placed in the situations above described, and who hope you particularly as a public man, as well as your correspondents, will endeavour to have the obnoxious parts of this Act removed.

---

*To the Editor of the London Medical and Physical Journal.*

Sir,—The critique on Mr. Carmichael's book in your last Number, contains a sort of suspicion that we in the north are so chaste as

 not not to be sufficiently acquainted with all the effects of illicit connections so common in London and Dublin. We are much obliged to you for your good opinion; but, how morally soever we may deserve it, the following extract from one of the last written inaugural dissertations will shew that the venerable Celsus, John Hunter, &c. even down to Sir James Fellows and Mr. Carmichael, have at last attracted our notice; and, I trust, you will admit that justice is done to each.

Had you been present at our Royal Medical Society during the last winter, you would have found the subject often introduced, and Hunter with Adams on Morbid Poisons, as familiar with us as Coke upon Littleton may be supposed to be among a similar group of law-students preparing themselves for the bar. But I need give you no better proof than the proposed extract from Dr. Burder's Disputation—I wish you had been present to hear him defend it. C. M.

*Edinburgh; Sept.* 5, 1815.

"Ingenium Johannis Hunterii præcellens animum medicorum ad morbos syphiloideos explorandos primum convertit. Symptomata abnormia, dentibus translatis se ostendentia, syphilitidis quidem similia, saepe autem sponte sua evanescentia, eum impulerunt suspicari, multos alios affectus morbidos, prout syphiliticos vulgo habitos, non esse. Conspectus hujus rei ab Hunterio primo habitus, commentatore ejus sagaci Doctore Adams, nec minus insigni ac erudito admiratore Abernethio fuit latius extentus. Multum notitiæ, si quæ alia, gratæ, quoque paulatim laboribus assiduis clarissimi Johannis Pearsonii, cujus facultates eximiæ et opportunitates extentissimæ modo singulari capacem operis aleæ tam difficilis reddunt. Diebus etiam recentioribus Dom. Carmichael materiem pretiosam curiosamque copiis scientiæ communibus addidit."

We feel much gratified for this communication from a new correspondent, on a subject which has been for some time confined almost to London, but which we are glad to see becomes, at last, matter of general examination.

We shall thankfully receive extracts from other inaugural dissertations, whatever may be their subject, which the authors or their friends may select as indicating the general tenor of the whole paper. Though it may be expected that such performances are, for the most part, of only temporary interest, yet, this is far from being always the case; witness the dissertation of Dr. Ludford, as well as several others, besides those which have been collected in the "Thesaurus." By the way, we conceive an additional volume or two, containing a new collection, might be very well received.

We are astonished to see a contemporary Journal repeat, from a German publication, the story of a venereal ulcer cured by arsenic, and concludes with a grave remark, that "the experiment *certainly* deserves to be repeated." Have we not heard the same of gold, of opium, of sarsaparilla, of acids, &c. &c. In short, till the accuracy of Mr. Hunter taught us better, had we ever ascertained tained what we meant by "venereal?" Our brother Journalists should have advised, in every future experiment, that the ulcer should be accurately described before the remedy is exhibited.

A society has lately been formed in Worcester, under the name of "The Medical and Surgical Society of Worcester," consisting of about fifty members, and including almost all the medical men of the city and county, which meets once a month. Its objects are the dissemination of medical knowledge amongst its members, the discussion of points of practice, and the keeping in the view of its members the principles of the profession. Each member, in his turn, gives in a subject of conversation, to which the attention of the society is confined for one or more meetings, according as the subject is more or less important and interesting. The institution admits of honorary and corresponding members, in the hope of receiving communications from medical men who reside at too great a distance to attend its meetings.

We are surprized to see no mention of a library in the above account.

---

We were preparing an abstract of the evidence concerning Bedlam, when we found the more prominent parts in the Times Newspaper. This, of course, lessened the more immediate interest in those events.

Pudet hæc opprobria nobis
Et dici potuisse & non potuisse refelli!

The length of Mr. Carmichael's papers, and our wish to offer as early as possible the very curious case of Mr. Highmore's Fœtus, and also our objection to the generally received theory upon it, must be our apology for the omission of other articles. Before our next is published, we are promised a Report, drawn up by the Committee of the House of Commons, on the Evidence concerning Bedlam.

---

The town of Brussels has recovered from the shock it first felt by the introduction of so many wounded soldiers in both armies. The mortality was much less than might have been expected, partly, perhaps, on account of time many of the wounded were left on the ground, and from the greater part of them being distributed in churches and other large buildings, instead of being crowded in hospitals and prisons.

All the learned of Paris are going to see an Hermaphrodite, who is shortly to make her appearance in London.

In our last we omitted to mention the promotion of Mr. Abernethy to the office of one of the senior Surgeons of St. Bartholomew's, after retaining that of Assistant-Surgeon for about thirty years. This vacancy was made by the resignation of Sir James Earle. Mr. Lawrence is appointed Assistant-Surgeon.

Dr. Southey, brother to the Laureat, as we expected, is chosen physician to the Middlesex Hospital.

Dr. Stewart, of Golden-square, has been elected Physician-Man-Midwife to the Westminster General Dispensary, in the room of Dr. S. Merriman resigned; and Dr. Merriman has been appointed the Consulting Physician-Man-Midwife.

METEO-

### METEOROLOGICAL REGISTER.

***From August the 25th, to September the 25th,* 1815.**

**Kept by C. BLUNT, Philosophical Instrument Maker, No. 38, Tavistock-Street, Covent-Garden.**

| Moon. | Day. | Wind. | Barometrical Pressure. Max. | Min. | Mean. | Temperature. Max. | Min. | Mean. | |
|---|---|---|---|---|---|---|---|---|---|
| | 26 | S | 30·05 | 29·99 | 30·03 | 75 | 50 | 64·66 | Fair |
| ◑ | 27 | S | 30· | 30· | 30· | 79 | 57 | 68·33 | Rain during the Night |
| | 28 | SW | 29·83 | 29 82 | 29·827 | 78 | 55 | 66·33 | Fair |
| | 29 | W by N | 29·98 | 29 90 | 29·94 | 76 | 46 | 62·33 | Fair |
| | 30 | W by N | 30·05 | 30·02 | 30·037 | 74 | 51 | 62· | Fair |
| | 31 | W | 30·09 | 30·04 | 30·067 | 71 | 49 | 61·75 | Fair |
| | 1 | W | 30·11 | 30· | 30·073 | 72 | 51 | 61·75 | Fair |
| | 2 | NW | 30·01 | 29·96 | 29·982 | 71 | 54 | 63· | Fair |
| ● | 3 | NW | 29·95 | 29·95 | 29·95 | 70 | 54 | 63· | Fair |
| | 4 | N | 29 97 | 29·88 | 29·922 | 66 | 50 | 61·5 | Fair |
| | 5 | NW | 30 03 | 29·97 | 29·99 | 65 | 40 | 56 5 | Fair |
| | 6 | N | 30·07 | 30·03 | 30·05 | 64 | 36 | 52·22 | Fair |
| | 7 | SW | 30·15 | 30·13 | 30·142 | 63 | 39 | 50·25 | Fair |
| | 8 | SW | 30·17 | 30·15 | 30·216 | 66 | 42 | 56· | Fair |
| | 9 | S | 30·16 | 30·13 | 30·142 | 69 | 43 | 55·75 | Fair |
| ◐ | 10 | S | 30·14 | 30·14 | 30·14 | 71 | 50 | 63·5 | Fair |
| | 11 | S | 30·14 | 30·11 | 30·127 | 71 | 48 | 62· | Fair |
| | 12 | S | 29·13 | 30 08 | 30·105 | 72 | 48 | 60·25 | Fair |
| | 13 | S | 29·99 | 29 93 | 29·952 | 80 | 47 | 65·5 | Fair, Fog in the Morning |
| | 14 | SE | 29·91 | 29·85 | 29·675 | 77 | 50 | 63 25 | Fair |
| | 15 | SE | 29·89 | 29·80 | 29·84 | 79 | 56 | 64·5 | Fair, Rain during Night |
| | 16 | S | 29·84 | 29·80 | 29·84 | 77 | 47 | 65· | Rain at Night |
| | 17 | S | 29·90 | 29·90 | 29·90 | 74 | 52 | 63· | Fair |
| ○ | 18 | SE | 30·17 | 30·08 | 30·122 | 74 | 53 | 64·5 | Fair |
| | 19 | SE | 30·19 | 30·12 | 30·147 | 70 | 45 | 57· | Fair |
| | 20 | SE | 30·07 | 30 | 30·032 | 67 | 40 | 56· | Fair |
| | 21 | E by S | 30· | 29·95 | 29·972 | 67 | 42 | 55· | Fair |
| | 22 | S | 29·81 | 29·76 | 29·78 | 65 | 46 | 57· | Rain |
| | 23 | SE | 29·68 | 29 64 | 29·655 | 63 | 43 | 54·25 | Rain at Night |
| | 24 | SW | 29·65 | 29·65 | 29 65 | 62 | 40 | 54· | Rain |
| | 25 | SW | 29·92 | 29·94 | 29·93 | 63 | 40 | 52·75 | Fair |

RESULTS.

| | | | | | |
|---|---|---|---|---|---|
| Mean barometrical pressure of the month | | 29·976 | Mean temperature of the month | | 60·095 deg. |
| Maximum 30·19, | wind at | SE | Maximum 80, | wind at | S |
| Minimum 29·64, | ——— | SE | Minimum 36, | ——— | N |

*Scale exhibiting the prevailing Winds during the Month.*

| N | NE | E | SE | S | SW | W | NW |
|---|---|---|---|---|---|---|---|
| 2 | 0 | 1 | 6 | 10 | 5 | 4 | 3 |

| | Mean barometrical pressure. | Mean temperature. |
|---|---|---|
| From the last quarter on the 27th Aug. to the new moon on the 3d of Sept. | 29·989 | 63·641 |
| ——— new moon on the 3d, to the first quarter on the 10th | 30·066 | 56·46 |
| ——— first quarter on the 10th, to the new moon on the 18th | 29·947 | 63·375 |
| From the full moon on the 18th, to the last quarter on the 26th | 29·911 | 56·312 |

### REPORT OF DISEASES.

Of the diseases which have fallen under our observation for this last month, Hooping Cough has been by far the most prevalent. In the district from which we write, nearly every house in two or three streets, chiefly inhabited by the poorer class of society, has been visited by this malady; the symptoms have differed only in their violence, and in their general resistance to our art. It has proved singularly fatal in spite of early local bleedings, emetics, purges, and blisters.

Cholera, slight Dysentery, and Diarrhœa, have been frequent. The former has usually yielded to small doses of carbonate of magnesia, diffused in mint-water; a blister to the region of the stomach has occasionally been requisite, as also a few drops of tincture of opium joined to the magnesia. The Dysenteric cases have been uniformly relieved by purging alternate mornings with sulphate of magnesia; and the Diarrhœas, by the cretaceous mixture, ipecacuan, and opium: a purgative having been in all cases previously administered.

A disease not very common (Pyrosis) is now under the Reporter's care. The patient is a married woman, about five and thirty years of age, and has been subject to the complaint, more or less, for these nine years; she has born several children, and menstruated regularly in the intervals; the symptoms are, in all respects, such as are so correctly described by Dr. Cullen in his First Lines. The usual stomachics and tonics having been tried in vain, she is now taking the oxyde of bismuth in the form and manner recommended by Dr. Marcet in cases of gastrodynia; she has taken it only a few days, and it has hitherto not produced any effect. Its failure or success will be noticed in our next Report.

A severe case of Hæmorrhagia Intestinalis, or, perhaps, Jecoris, occurring in a female of fifty, of rather intemperate habits, was cured in a few days, by aperients given every morning till natural evacuations took place. The quantity of blood discharged for the first two days, were nearly two quarts of a dark colour; her strength was supported by beef tea.

Rheumatism, in its acute form, and which was unusually prevalent in the spring, still continues to occur. Few of the cases have required bleeding, and the majority of them have had their inflammatory symptoms removed by frequent and copious purging, assisted by an antiphlogistic regimen, and keeping the patients, as in purely febrile affections, in a cool state.

Six cases of Fever have occurred, three of which appeared, from the cord-like feel of the artery, to be purely inflammatory. The existence of synocha, independent of organic affection, has been denied; yet, in the instances above-mentioned, neither the brain nor any of the thoracic or abdominal viscera were perceptibly affected. The subjects were young (from sixteen to thirty-five), and were cured by saline purgatives, having their action quickened by jalapæ pulvere, exhibited every other day, so as to purge freely. The lancet was not had recourse to on account of there being no determination to any organ in particular, but every part appearing to suffer equally. Of the three others, one was what would have been termed Typhus gravior, the others Synochus. The subject of the former was a seaman, aged forty, who had been labouring under the disease, when we first saw him, about ten days. He had been lately paid off, and indulging freely in the use of spirits. He had been treated with stimulants, such as camphor, æther, wine, &c. The tongue, mouth, and lips, were much parched, and of a dark mahogany colour; he was delirious, his pulse firm, though not strong; floccitatio; urine small in quantity and high coloured; alvine evacuations few, and those passed involuntarily. He was directed to be kept cool, no stimuli administered, and to take every six hours a gently aperient saline medicine. He recovered under this mode of treatment. The latter shewed nothing unusual, and were soon recovered from, by the purgative practice.

Head-aches, attended with giddiness and dimness of sight, have been very common, both in men and women of middle age, and, though they have been generally relieved by cupping, purging, and blistering, the relief afforded has only been temporary. Bleeding from the arm was seldom employed, from having long since remarked, that, unless the disease be of an acute nature, few patients bear well repetitions of the operation.

